# Genomic Analyses of Three Malaria Vectors Reveals Extensive Shared Polymorphism but Contrasting Population Histories

**DOI:** 10.1093/molbev/msu040

**Published:** 2014-01-09

**Authors:** Samantha M. O’Loughlin, Stephen Magesa, Charles Mbogo, Franklin Mosha, Janet Midega, Susan Lomas, Austin Burt

**Affiliations:** ^1^Department of Life Sciences, Imperial College London, Silwood Park, Ascot, United Kingdom; ^2^NIMR Amani Research Centre, Muheza, Tanzania; ^3^RTI International, Global Health Division, Dar es Salaam, Tanzania; ^4^Kenya Medical Research Institute, Centre for Geographic Medicine Research Coast, Kilifi, Kenya; ^5^Malaria Public Health Department, Centre for Geographic Medicine, KEMRI-Wellcome Trust Research Programme, Kenyatta National Hospital Grounds, Nairobi, Kenya; ^6^Kilimanjaro Christian Medical University College, Moshi, Tanzania; ^7^Department of Life Sciences, Imperial College London, South Kensington Campus, London, United Kingdom

**Keywords:** RADseq, *Anopheles gambiae*, population genomics, 2La, *Anopheles arabiensis*, *Anopheles merus*

## Abstract

*Anopheles gambiae s.l.* are important malaria vectors, but little is known about their genomic variation in the wild. Here, we present inter- and intraspecies analysis of genome-wide RADseq data, in three *Anopheles gambiae s.l.* species collected from East Africa. The mosquitoes fall into three genotypic clusters representing described species (*A. gambiae*, *A. arabiensis*, and *A. merus*) with no evidence of cryptic breeding units. *Anopheles merus* is the most divergent of the three species, supporting a recent new phylogeny based on chromosomal inversions. Even though the species clusters are well separated, there is extensive shared polymorphism, particularly between *A. gambiae* and *A. arabiensis*. Divergence between *A. gambiae* and *A. arabiensis* does not vary across the autosomes but is higher in X-linked inversions than elsewhere on X or on the autosomes, consistent with the suggestion that this inversion (or a gene within it) is important in reproductive isolation between the species. The 2La/2L+^a^ inversion shows no more evidence of introgression between *A. gambiae* and *A. arabiensis* than the rest of the autosomes. Population differentiation within *A. gambiae* and *A. arabiensis* is weak over approximately 190–270 km, implying no strong barriers to dispersal. Analysis of Tajima’s *D* and the allele frequency spectrum is consistent with modest population increases in *A. arabiensis* and *A. merus*, but a more complex demographic history of expansion followed by contraction in *A. gambiae.* Although they are less than 200 km apart, the two *A. gambiae* populations show evidence of different demographic histories.

## Introduction

In their role as vectors of malaria, *Anopheles* mosquitoes were indirectly responsible for an estimated 600,000 deaths in sub-Saharan Africa in 2010 (World Health Organization 2012). Knowledge of the genetics of wild populations of these mosquitoes is important for both conventional and novel vector control methods. Conventional control of vectors by insecticide-treated bed nets and indoor residual spraying can be hampered by the spread of insecticide resistance (Enayati and Hemingway 2010), and in the case of novel control methods such as sterile insect technique (SIT) and introduction of transgenes, a knowledge of the size of mosquito populations (which can be estimated indirectly by genetic diversity) and population structure will be required (James 2005). Studying the genetics of wild populations can also uncover the existence of cryptic species, which may differ in their vectorial capacity (Riehle et al. 2011).

In East Africa, the main vector species are the closely related members of the *Anopheles gambiae s.l.* species complex and the less closely related *A. funestus*. Within *A. gambiae s.l.*, the most important vectors are the widespread species *A. gambiae* and *A. arabiensis*, although all other members of the complex are also malaria vectors, with the exception of *A. quadriannulatus* and *A. amharicus* (formerly *A. quadriannulatus* B, Coetzee et al. 2013)*.* All species within the complex will produce fertile female offspring in laboratory crosses (Davidson 1964), but with the exception of *A. gambiae* × *A. coluzzii* (formerly *A. gambiae* S and M form, Coetzee et al. 2013); male offspring are infertile.

Resolving the evolutionary relationships of the species within the complex has proved problematic. In early chromosomal studies, it was assumed that the nonvector species (*A. quadriannulatus*) was basal, and the “standard” inversion nomenclature was given to the inversions in this species. *Anopheles gambiae* and *A. merus* were identified as sibling species due to two shared fixed inversions on the X chromosome (designated Xag; *A. arabiensis* has several different fixed inversions designated Xbcd). Relationships between species could mostly be parsimoniously explained from fixed and polymorphic inversions, with the exception of inversion 2La. To explain how this inversion could be segregating in *A. gambiae* but fixed in the inverted form in *A. arabiensis* and *A. merus*, it would either have to have arisen multiple times independently or have arisen twice (in *A. arabiensis* and *A. merus* separately) and transferred between *A. gambiae* and *A. arabiensis* by introgressive hybridization (Coluzzi et al. 1979). An early study supported the independent origin of the inversion in *A. merus*, designated 2La′ (Caccone et al. 1998). Later, when DNA sequence data became available, it was apparent that *A. gambiae* and *A. arabiensis* showed high sequence similarity and were nonmonophyletic at many markers, including mitochondrial DNA (mtDNA) (Besansky et al. 1994, 1997, 2003). These studies included samples from colonies (Besansky et al. 1994) and from West and East Africa (Besansky et al. 1997, 2003). This low divergence between *A. gambiae* and *A. arabiensis* was attributed to introgression between the two species (rather than retained ancestral polymorphism), and it was suggested (based on samples from both West and East Africa) that 2La had passed from *A. arabiensis* into *A. gambiae*, conferring adaptation to more arid habitats (Coluzzi et al. 1979, 1985; Besansky et al. 1994, 1997, 2003; Donnelly et al. 2004). Subsequently, more comprehensive analysis of 2La indicated that the inversion had a single origin and that the 2La orientation was ancestral (Ayala and Coluzzi 2005; Sharakhov et al. 2006). Recently, a new analysis of chromosomal inversions using an outgroup has placed *A. gambiae* and *A. merus* as basal in the phylogeny (Kamali et al. 2012).

Until now, most studies of the population structure of wild *A. gambiae* have used mtDNA and/or small numbers of microsatellites and nuclear gene sequences. *Anopheles gambiae* and, to a lesser extent, *A. arabiensis* exhibit evidence at mtDNA and microsatellites of recent populations expansion (Besansky et al. 1997; Donnelly et al. 2001). As both species are associated with humans and human habitation, it is likely that they have experienced extensive population and range expansions along with the spread of humans. The genetic signal left by such expansions can lead to overestimation of current gene flow by obscuring population structure (Besansky et al. 1997; Donnelly et al. 2001). This would explain why most studies have found high inferred gene flow in both species even over very long distances, despite the limited dispersal ability of these organisms (Besansky et al. 1997; Kamau et al 1999; Pinto et al. 2013). There is some evidence that physical barriers such as the Rift Valley and water (ocean) can be a barrier to gene flow in *A. gambiae* (Lehmann et al. 1999, 2000; Kayondo et al. 2005; Moreno et al. 2007). *Anopheles arabiensis* have generally shown less population structure than *A. gambiae* (e.g., Kamau et al. 1999; Simard et al 2000; Nyanjom et al. 2003), although there have been some exceptions; most notably a recent microsatellite study in southern Tanzania found the reverse to be true, with no population structure found in *A. gambiae* but high levels in *A. arabiensis* (Ng’habi et al. 2011).

Studies using mtDNA suffer from the inherent drawback that it is essentially a single locus. Microsatellites give a multilocus picture, avoiding the danger of misinterpreting selection for demography but have the disadvantages of limited numbers (the maximum used in *A. gambiae* studies is 17 in Kayondo et al. 2005), ascertainment bias, and an ill-defined mechanism of evolution that makes some analyses (such as inferences of population history) difficult. In the study of natural populations of *A. gambiae*, studies using genome-wide single-nucleotide polymorphisms (SNPs) so far have concentrated on the relationship between *A. gambiae* and *A. coluzzi* in West Africa and have used microarrays rather than next-generation sequencing, therefore could not measure genetic diversity within species (Neafsey et al. 2010; Weetman et al. 2011). The former study identified regions of high divergence between *A. gambiae* and *A. coluzzii* from Mali and also included analysis of a pool of *A. arabiensis* from Burkina Faso; divergence was found to be fairly homogenous across the genome between *A. gambiae*/*coluzzii* and *A. arabiensis*, except for low divergence at 2La and high divergence across the X chromosome (Neafsey et al. 2010). In PCA analysis of 2La SNPs, *A. gambiae*/*coluzzii* with the 2La/2La karyotype clustered with *A. arabiensis*, supporting the hypothesis of 2La introgression between species. The study by Weetman et al. (2011) identified major effect insecticide resistance SNPs in wild *A. gambiae* and *A. coluzzii* from Ghana and Cameroon, using a customized SNP array enriched for known insecticide resistance loci. A single study has used next-generation whole-genome resequencing of pooled, wild-caught *A. gambiae* of known 2La karyotypes to investigate population divergence along a cline of the 2La inversion in Cameroon (Cheng et al. 2012); they found patterns consistent with gene flux, migration, and natural selection and identified several candidates for genes associated with environmental variables.

Here, we use restriction-site associated DNA sequencing (RADseq) to genotype genome-wide markers in individual wild-caught *A. gambiae*, *A. Arabiensis*, and *A. merus*. RADseq exploits next-generation sequencing methods to sequence a small, random but reproducible fraction of the genome, allowing multiplexing of samples and high-depth sequencing at an economical cost (Baird et al. 2008). RADseq avoids much of the ascertainment bias which would be inherent in a microarray approach. Our samples were sourced from three sites in East Africa (fig. 6); *A. gambiae* were collected from two locations approximately 200 km apart (Kilifi [Kenya] and Muheza [Tanzania]), *A. arabiensis* from three locations approximately 200 km apart (Kilifi [Kenya], Muheza and Moshi [Tanzania]), and *A. merus* from one location (Kilifi [Kenya]). We show that RADseq libraries can successfully be created from single mosquitoes, with little contamination from blood meals or microorganisms. We aligned the RADseq reads to the *A. gambiae* PEST genome and report genetic diversity metrics of the three species. We have used the RADseq SNPs to investigate whether divergence between the three species is consistent with the recently published phylogeny based on chromosomal inversions. We look for heterogeneity in diversity and divergence in different regions of the genome, particularly at known inversions. We investigate whether genome-wide SNPs reveal a similar low level of population structure to that seen in previous studies and use allele frequency spectra to compare demographic history between collection sites and species.

## Results and Discussion

### Summary of RADseq Data

A summary of sequence read and alignment statistics are given in the supplementary results, Supplementary Material online.

In addition to filtering sites based on quality of the base calls (i.e., phred score ≥ 25, coverage ≥ 15×, location ≥ 7 bp from an indel, see Materials and Methods), we also wanted to avoid biases that may be exacerbated by including missing data (Arnold et al. 2013; Gautier et al. 2013), and therefore filtered out all sites where any sample had missing data. As a result, different analyses are based on different numbers of sites, depending on the number of samples included in the data set (table 1). This is partly due to divergence of *A. arabiensis* and *A. merus* from the PEST genome but also due to polymorphisms in the genomes at the restriction sites; the more samples and species that are included, the more likely a RAD tag will be absent in one or more samples.

**Table 1.** Metrics of Data Sets Containing Variant and Invariant Sites.

Data Set	Species Included	No. Indivs.	No. of Tag Locations	No. bp	Mean Coverage	Mean Distance between Tags (kb)	No. of SNPs^a^	Mean (Max) SNPs Per Tag
1	All samples	72	1,292	64,556	108	200	4,896 (5,008)	3.79 (19)
2	*A. gambiae* and *A. arabiensis*	60	1,610	87,504	101	162	5,611 (5,681)	3.34 (23)
3	*A. gambiae*	24	2,033	172,655	88	131	6,387 (6,437)	3.14 (17)
4	*A. arabiensis*	36	2,049	129,315	106	130	5,137 (5,195)	2.51 (23)
5	*A. merus*	12	2,280	253,043	134	115	4,135 (4,161)	1.81 (13)

^a^Number in brackets shows number of SNPs in variant only data sets. Bayesian variant calling resulted in slightly different numbers of SNPs in variant only vs. genotype data sets.

The majority (88%) of SNPs were in noncoding regions, and 55% of SNPs in exons were synonymous. The proportion of reads aligning to exons was about twice as high for the X chromosome as for the autosomes (27% vs. 13%, 40% vs. 17%, and 43% vs. 19% for *A*. *gambiae*, *A. arabiensis*, and *A. merus*, respectively). This pronounced increase (particularly in *A. merus*) is due to the high divergence of the X chromosome between species, meaning that RAD tags that align to PEST are more likely to be confined to exons where variation is constrained.

### Identification of Breeding Units and Species Divergence

**Fig. 1.**
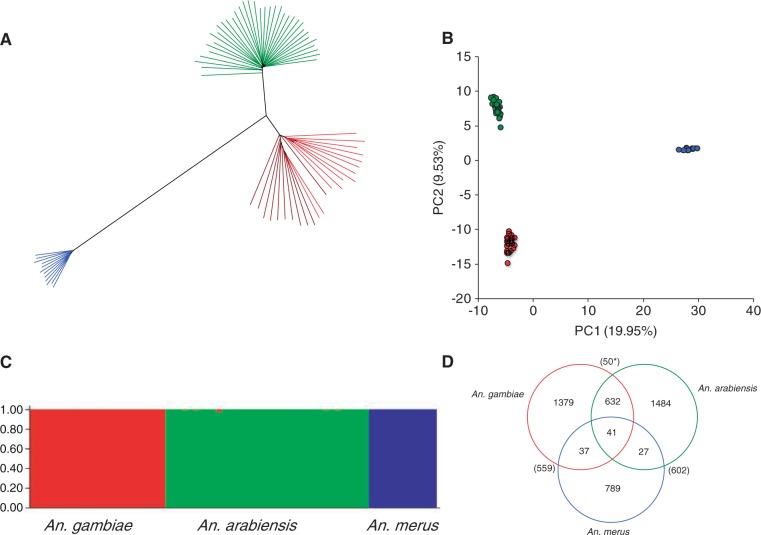
Interspecies relationships. (*A*) Unrooted neighbor-joining tree based on average pairwise distances (*D*_XY_), using 4,711 autosomal SNPs. (*B*) Principal components analysis of 847 autosomal SNPs (one SNP chosen randomly per tag location). (*C*) Results from a Bayesian cluster analysis of one SNP per tag location using STRUCTURE. The highest log-likelihood was for three populations (*k* = 1–5 tested). The three populations corresponded to the three species, and each individual was assigned almost entirely to one population, with only one exception (KA11, see text and supplementary results, Supplementary Material online, for further details). Each individual is represented by a vertical bar. Analysis carried out using one SNP per RAD tag location (913 genome-wide SNPs). (*D*) Venn diagram showing number of private and shared polymorphisms for the whole genome. Also shown in brackets are the numbers of fixed differences between the species. *Fixed differences between *Anopheles gambiae* and *A. arabiensis* increase to 60 when sample KA11 is excluded. Color scheme: red = *A. gambiae* (in the phylogeny, light red = Kilifi, dark red = Muheza), green = *A. arabiensis*, blue = *A. merus*. All analyses are from SNP set 1.

High-density multilocus genotypes such as given by RADseq analysis should allow detection of previously undescribed taxa not included in the standard rDNA identification test (such as that recently found by Riehle et al. 2011). To test for such taxa, we constructed a neighbor-joining tree and performed both principal components and STRUCTURE analyses (fig. 1*A*–*C*). In all three analyses, the individuals fall into three distinct groups, with no suggestion of any cryptic taxon. In two instances, the clustering differed from our initial rDNA identification; repeating the latter showed that these two individuals had been misidentified initially.

Though the individuals fall into three distinct groups, nevertheless there are many polymorphisms that are shared between two of the species or even all three species (fig. 1*D* and supplementary fig. S5 sliding window, Supplementary Material online). Between *A. gambiae* and *A. arabiensis*, shared polymorphisms outnumber fixed differences by more than 13:1. Though distinct in a genome-wide analysis, the three species are not genealogically independent: it is not the case that all alleles in one species are more closely related to other alleles in the same species than to alleles in a different species.

These analyses also indicate that, of the three species, *A. gambiae* and *A. arabiensis* are the most similar, with *A. merus* more distantly related. The same result is also seen in calculations of *F*_ST_ (*F*_ST_ = 0.40, 0.71, and 0.74 for *gambiae*–*arabiensis*, *gambiae*–*merus*, and *arabiensis*–*merus*, respectively). It has previously been suggested that *A. gambiae* and *A. merus* are sister species, because they share a particular gene order on the X chromosome, which was thought to have been derived via two inversions from a gene order seen elsewhere in the species complex (the Xag inversion; Coluzzi et al 1979; Garcia et al. 1996). Recently, Kamali et al. (2012) performed a parsimony analysis of chromosomal rearrangements and proposed the alternative hypothesis that the *gambiae*–*merus* gene order is ancestral in the species complex, and furthermore that *A. merus* is the most basal species (i.e., is a sister group to a clade containing all other species). Our finding that *A. merus* is the most divergent of the three species at the nucleotide level is fully consistent with this suggestion.

**Fig. 2.**
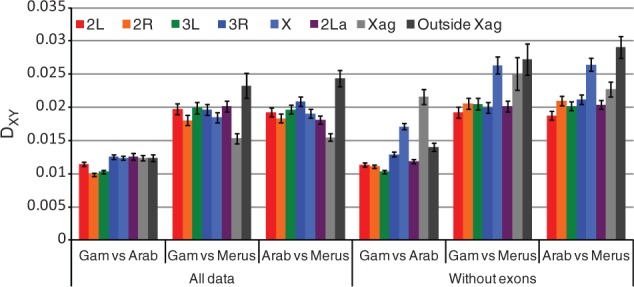
Divergence (as measured by *D*_XY_) between different species pairs, for different regions of the genome. Data set 1, with and without exons. Error bars show ± 1 SD.

Interestingly, if one looks only at the region of the X chromosome where the *gambiae*–*merus* gene order is unique (i.e., within Xag), *A. gambiae* and *A. arabiensis* are still the most similar pair of species, but the difference is not as great as elsewhere in the genome (the ratio of *gambiae*–*arabiensis* divergence to the average of *gambiae*–*merus* and *arabiensis*–*merus* divergence is 0.43 for autosomes, 0.68 for Xag, and 0.36 for the rest of X; difference between autosomes and Xag is significant by *t*-test, *P* = 0.01; figs. 2 and 5). Note that we are measuring absolute divergence between species (*D*_XY_) rather than relative divergence (*F*_ST_ or *D*_A_), so our results are not influenced by diversity within species. Because of the bias in our X chromosome data toward the more constrained exons, we also calculated divergence for the noncoding regions alone; this increased all pairwise comparisons on the X chromosome but had little or no effect on autosomal divergence (fig. 2). The order of X chromosome divergence between species remained the same, but the divergence between *A. gambiae* and *A. arabiensis* at Xag became more pronounced. One possible explanation is that the inversion (or a speciation gene within it) has acted as a barrier to gene flow between *A. gambiae* and *A. arabiensis*, and other regions of the genome either took longer to stop transferring between species or continue to transfer now at a higher rate. Consistent with this idea, crosses and back-crosses of *A. gambiae* and *A. arabiensis* in the laboratory have shown that introgression of the X chromosome is much more difficult (i.e., associated with greater sterility and lethality) than for the autosomes (Della Torre et al. 1997; Slotman et al. 2005). The same lab crosses found that chromosome 2 introgresses somewhat easier than chromosome 3, but we see no obvious difference in divergence between these two chromosomes (*t*-test of nonoverlapping sliding window of *D*_XY_ = 1.22, *P* = 0.23).

One *A. arabiensis* sample showed an unusual level of shared polymorphisms on chromosomes 2R (25 SNPs) and X (28 SNPs). Discounting experimental error, it is possible that this *A. arabiensis* individual carried some *A. gambiae* alleles from a past hybridization. More details on this sample are given in supplementary results, Supplementary Material online.

### The 2La Inversion

**Fig. 3.**
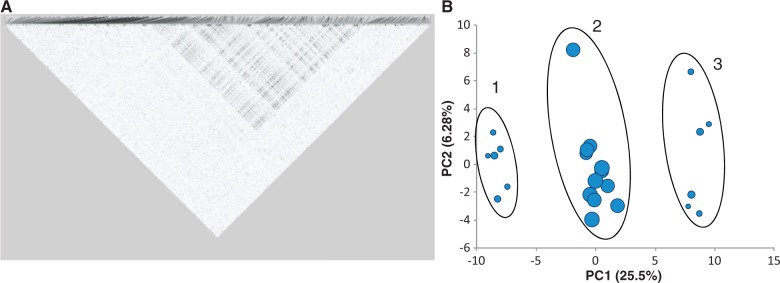
(*A*) Grid plot of LD *r*^2^ for *Anopheles gambiae* chromosome 2L (*N* = 24) and (*B*) PCA plot of 156 SNPs within the 2L+^a^ inversion. The circles are scaled to the level of average heterozygosity for each sample. The location of the region of high LD corresponds to the known chromosomal coordinates of 2L+^a^, e.g., those published on Vectorbase. SNP set 3.

**Fig. 4.**
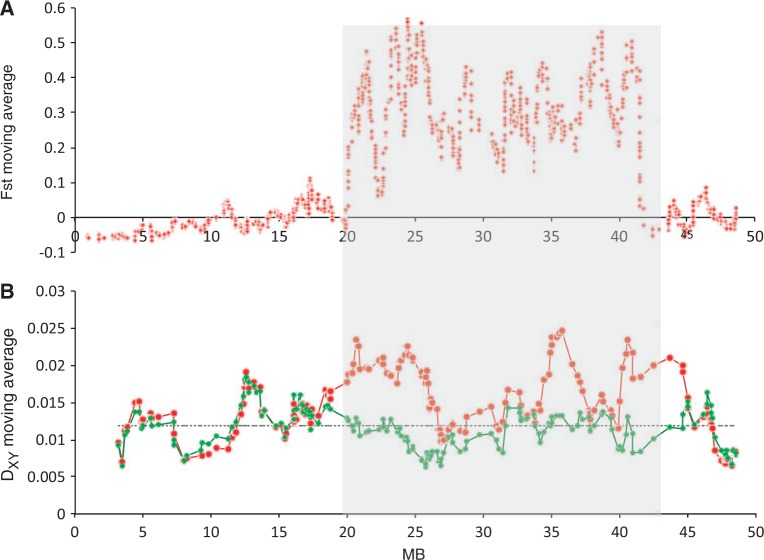
Sliding windows of *F*_ST_ and *D*_XY_ between inversions. Shaded area denotes location of 2L+^a^ inversion. (*A*) *Anopheles gambiae* 2La/a and 2L+/+ samples, across chromosome 2L (1,489 SNPs). *F*_ST_ measured locus-by-locus and averaged in a 25 SNP window moving in 1 SNP steps. Average *F*_ST_ for 2La = 0.567. SNP set 3. (*B*) Average pairwise nucleotide divergence (*D*_XY_) across chromosome 2L (18,065 sites) for *A. gambiae* and *A. arabiensis*. 1,000 site sliding window in 100 site steps. Red: *D*_XY_ between *A. gambiae* 2L+/+ homokaryotype samples and *A. arabiensis*; Green: *D*_XY_ between *A. gambiae* 2La/a homokaryotype samples and *A. arabiensis*. Gray dotted line shows autosome average *D*_XY_. Data set 2.

If inversions are segregating in a population, then rates of recombination between chromosomes with different orientations are reduced, particularly near the inversion breakpoints, resulting in an increase in linkage disequilibrium (LD, defined as associations between alleles at different loci). LD plots clearly show that our *A. gambiae* samples were segregating for the well-known 2La inversion (fig. 3*A*). Principal components analysis (PCA) of 156 SNPs within the 2La inversion shows three clear clusters, presumably corresponding to samples containing the two homokaryotypes and the heterokaryotype (fig. 3*B*). As expected, samples within the putative heterokaryotype cluster showed higher heterozygosity than those in the two homokaryotype clusters (fig. 3*B*), and *F*_ST_ between the two homokaryotypic clusters is high in the inversion but drops sharply away outside it (fig. 4*A*). Although clearly distinct, the two inversion homokaryotypes share 36 polymorphisms, indicating some level of gene flow between them (presumably due to gene conversion and double crossing-over [Stump et al. 2007]). The frequency of the two chromosomal orientations did not differ between Muheza and Kilifi (χ^2^ probability = 0.24) and showed no obvious deviation from Hardy–Weinberg proportions (χ^2^ probability = 1.0).

**Fig. 5.**
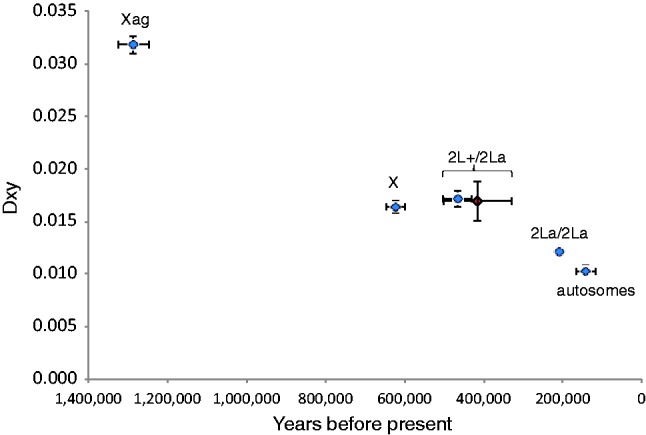
Noncoding divergence between *A. gambiae* and *A. arabiensis* at different parts of the genome. *x* axis: time from present calculated from net pairwise divergence (*D*_A_) using *D*_A_ = 2*µT* (see Materials and Methods for value of *µ*); *y* axis = *D*_XY_. Data points: Xag (2,234 sites), X outside Xag (2,006 sites), 2La (8,475 sites), and autosomes excluding 2La (58,752 sites). Data excludes exons. Blue diamonds represent comparisons between *A. arabiensis* and *A. gambiae*. The red diamond represents the comparison between the two 2La homokaryotypes in *A. gambiae*. Data set 2.

In PCA of SNPs within the 2La inversion, *A. arabiensis* samples do not fall into a cluster with *A. gambiae* 2La/2La samples (supplementary fig. S6, Supplementary Material online), contrasting with the microarray analysis of West African *A. gambiae*/*coluzzii* and *A. arabiensis* (Neafsey et al. 2010). It has previously been suggested that the 2La inversion introgressed relatively recently from *A. arabiensis* (which is fixed for that arrangement) into *A. gambiae*, and that this was important in expanding the range of habitats in which *A. gambiae* can persist (Coluzzi et al. 1979; Besansky et al. 2003; White et al. 2007). If so, then one would expect divergence between *A. arabiensis* and one of the two homokaryotypes to be relatively low. However, we find instead that the divergence between *A. arabiensis* and one of the homokaryotypes inside the inversion is about the same as divergence outside the inversion (and the rest of the genome), whereas divergence with the other homokaryotype is higher inside the inversion (especially near the inversion breakpoints) than elsewhere (figs. 4 and 5). Moreover, if the 2La inversion has recently introgressed from *A. arabiensis* to *A. gambiae*, one would expect the *A. gambiae* homokaryotype that is more similar to *A. arabiensis* (i.e., 2La/2La) to be less polymorphic than the homokaryotype that is more divergent (i.e., 2L+^a^/2L+^a^), but this is not the case (*t*-test of nucleotide diversity [π] within 2La between each homokaryotype nonsignificant; see also White et al. 2007), and we would also expect π within the homokaryotypes to be lower than the rest of the genome (as found in a West African colony mosquitoes in Mathiopoulos and Lanzaro 1995), which was also not the case (*t*-test of π within the homokaryotypes vs. collinear autosomal π nonsignificant). These results do not suggest 2La has introgressed from *A. arabiensis* to *A. gambiae* (or at least no more than other autosomal regions). One explanation for our findings is that the 2La to 2L+^a^ inversion event occurred prior to the split between *A. gambiae* and *A. arabiensis*, and the inversion has remained polymorphic in *A. gambiae*, while 2L+^a^ was lost in *A. arabiensis*. Note, though, that even the inversion breakpoints are not as divergent as Xag (figs. 4 and 5). It will be interesting to see whether SNP analysis of *A. gambiae* and *A. coluzzii* collected from West Africa reveal the same divergence of 2La karyotypes relative to *A. arabiensis* or whether they show the patterns predicted above which would support the hypothesis of introgression.

### Other Segregating Inversions

No other inversions appeared to be segregating in our *A. gambiae* sample (supplementary fig. S7, Supplementary Material online). *Anopheles gambiae* has been reported to be polymorphic for 2Rb in Kenya (about 18% frequency) and Tanzania (5% frequency) (Mnzava and Di Deco 1990; Petrarca and Beier 1992). In our *A. gambiae* samples, we do not see any regions of high LD on 2R, and PCA of SNPs within the 2Rb region show no clustering (data not shown), suggesting there is only one inversion type present, although cytogenetic karyotyping would be necessary to confirm this.

LD plots for *A. arabiensis* indicate that our samples are segregating for two inversions, 2Rb and 3Ra (supplementary fig. S8, Supplementary Material online; Coluzzi et al. 1979). PCA of the region covered by 2Rb shows that there are three clusters (plus one outlier further discussed in the text), again with the middle one having a higher average heterozygosity and *F*_ST_ between the homokaryotypes elevated within the inversion (supplementary fig. S9, Supplementary Material online). For 3Ra, there are only two clusters in the PCA plot, one of which has higher heterozygosity than the other, indicating that one of the homokaryotypes was not included in our sample. In western Kenya, 3Ra occurs at a frequency of about 5% (Petrarca and Beier 1992) and in Tanzania it has been reported at frequency from 0% to 15% (Mnzava and Di Deco 1990); this is very close to the 7% minor allele frequency that we see in our data, suggesting that the main cluster represents individuals with the standard 3R+^a^. Neither the 2Rb nor 3Ra inversions are correlated to sampling location, nor depart from Hardy–Weinberg equilibrium frequencies.

There were no regions of elevated LD apparent in the *A. merus* sample (supplementary fig. S11, Supplementary Material online). This is not surprising, as no inversions have yet been reported to be segregating in this species (Petrarca et al. 1984).

### Population Differentiation within Species

**Fig. 6.**
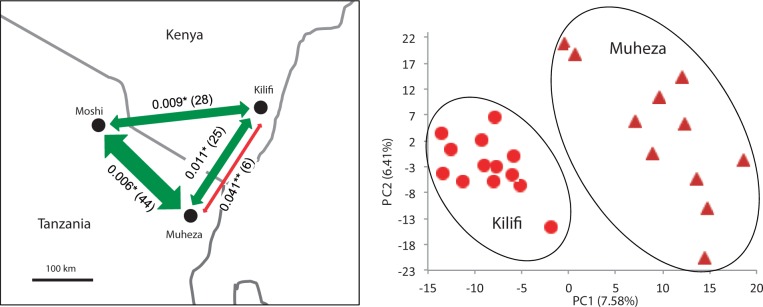
Population structure of *A. gambiae* and *A. arabiensis*. Left panel: *F*_ST_ and migration (Nm) between sample sites. Green arrows connect *A. arabiensis* populations, red arrow *A. gambiae* populations. *P < 0.05, **P < 0.0001. Data sets 3 and 4. Right panel: PCA of *A. gambiae* SNPs. PCA on one randomly chosen SNP per tag, all tags (1,593 SNPs). Light red circles = samples from Kilifi, Kenya, dark red triangles = samples from Muheza, Tanzania. PCA results were consistent across three data sets of one random SNP per tag. Data set 3.

Previous studies analyzing mtDNA and microsatellite loci have typically found only weak geographical differentiation for both *A. gambiae* (e.g., *F*_ST_ of 0.036 over 6,000 km and *F*_ST_ of 0.024 over 1,340 km [Lehmann et al. 1996, 2003]) and *A. arabiensis* (e.g., *F*_ST_ of 0.012 over 250 km [Simard et al. 2000]). Using genome-wide SNPs, we also find weak differentiation, extending across the genome, indicating that it is caused by demography rather than selection (supplementary fig. S12 sliding window of *F*_ST_, Supplementary Material online,). Kilifi and Muheza are separated by 190 km, and while PCA and STRUCTURE analysis of 1,593 genome-wide SNPs in *A. gambiae* (one SNP chosen randomly per tag) does show the populations as distinct (fig. 6), the difference in allele frequencies between the two populations is small (*F*_ST_ = 0.041, *P* < 0.0001). *Anopheles arabiensis* showed even less differentiation among the three sites at which it was collected (separated by 190–270 km, *F*_ST_ ranging from 0.006 to 0.01), with no clustering in PCA or STRUCTURE (results not shown). As a result, the inferred level of gene flow between populations is higher for *A. arabiensis* than for *A. gambiae* (fig. 6). This result is opposite to that found in the Kilombero Valley of southern Tanzania, where microsatellite *F*_ST_ for *A. arabiensis* tended to be larger than those for *A. gambiae* (0.006–0.1 vs. 0.003–0.01, respectively [Ng'habi et al. 2011]). No microgeographic population structure was identified within sampling locations in any species (see supplementary results, Supplementary Material online).

In both *A. gambiae* and *A. arabiensis*, differentiation as measured by *F*_ST_ was higher for the X chromosome than for the autosomes, although this was only significant for *A. gambiae* (*t*-test *P* = 0.024; supplementary table S2, Supplementary Material online). The X chromosome has three-fourth the effective population size than autosomes, so will be more affected by genetic drift; therefore under neutrality, we would expect to see higher relative divergence within species at the X chromosome.

If samples derived from separate populations are analyzed together, and the populations differ in allele frequency, then a deficiency of heterozygotes compared with Hardy–Weinberg proportions may be expected (the “Wahlund effect”). No overall deviation from Hardy–Weinberg proportions was detected in any population except for *A. arabiensis* from Kilifi (*F*_IS_ = 0.11, *P* = 0.0057). The deficit is consistent across one SNP per tag data sets and across all chromosomes except X. The cause of this deviation is not clear: the high *F*_IS_ is not associated with inversions and is not caused by population structure (*k* = 1 in STRUCTURE), so it may be caused by inbreeding.

### Nucleotide Diversity and Population History

This is the first study of a genome-wide sample of sequences in wild-caught *A. gambiae s.l*. Previously, some nuclear genes from wild mosquitoes have been sequenced, and noncoding nucleotide diversity (π) was in the order of 0.0013–0.04 (Mukabayire et al. 2001; Besansky et al. 2003; Obbard et al. 2007), with lower diversity in *A. arabiensis* and *A. merus* than in *A. gambiae* (Besansky et al. 2003). Autosomal nucleotide diversity in our data set varied approximately 2-fold, being lowest in *A. merus* (*π* = 0.0041, *θ*_W_ = 0.0044) and highest for *A. gambiae* in Muheza (*π* = 0.0085, *θ*_W_ = 0.0088; table 2). This is not an unbiased measure of diversity (particularly in *A. arabiensis* and *A. merus*) due to aligning with the PEST reference genome, which was necessary to obtain genomic locations of our markers and for interspecies analysis. We have limited the bias by creating the single species data sets (data sets 3, 4, and 5). Some downward bias of diversity will also be introduced by the RADseq method (discussed in more detail later in the text). A detailed breakdown of overall and noncoding π per population and data set is given in supplementary figure S13, Supplementary Material online.

**Table 2.** Diversity Indices for *Anopheles gambiae, A. arabiensis,* and *A. merus*.

			Genotype Data Set					SNP Set	
Species	Location	*N*	No. of Informative Sites	*S*	*π* ± SD	*θ*_W_	Tajima’s *D*	*S*	Fis
*A. gambiae*^a^	Kilifi	13	172,655	4,852	8.07 ± 0.10	7.36	0.3840	4,890	0.0628^b^
	Muheza	11	172,655	5,510	8.47 ± 0.13	8.75	−0.1357	5,556	0.0474^b^
	All	24	172,655	6,387	8.42 ± 0.06	8.34	0.0377	6,437	0.0555*
*A. arabiensis*^c^	Kilifi	11	129,315	3,542	6.85 ± 0.13	7.51	−0.3650	3,582	0.1073*
	Muheza	13	129,315	3,692	6.80 ± 0.09	7.48	−0.3674	3,736	0.0220^b^
	Moshi	12	129,315	3,639	6.76 ± 0.10	7.54	−0.4203	3,736	0.0256^b^
	All	36	129,315	5,137	6.84 ± 0.04	8.20	−0.5846	5,195	0.0534*
*A. merus*^d^	Kilifi	12	253,043	4,135	4.13 ± 0.06	4.38	−0.2255	4,161	0.0284^b^

Note.—Genotype data set: includes invariant and variant sites, no missing data. SNP set: variant sites only, no missing data. *N* = number of samples; *S* = number of segregating sites. Fis tested for significance by 8,000 permutations. *π* and *θ*_W_ are per site and have been multiplied by 10^3^.

^a^From data set 3.

^b^Not significant.

^c^From data set 4.

^d^From data set 5.

*P < 0.05.

Within *A. gambiae*, the Muheza population is slightly but significantly more diverse than Kilifi (paired *t*-test across chromosome arms; *P* = 0.004 and 0.001 for π and θ_W_, respectively), whereas there were no significant differences in nucleotide diversity among the three populations of *A. arabiensis*. Under a standard neutral model, π and θ_W_ are estimates of 4*N*_e_*μ*, where *N*_e_ is the effective population size and *μ* is the mutation rate. Using a mutation rate of 1.1 × 10^−^^9^ per generation (see Methods and Materials), N_e_ was approximately 1 million for *A. merus* and 2 million for *A. gambiae* and *A. arabiensis*.

**Fig. 7.**
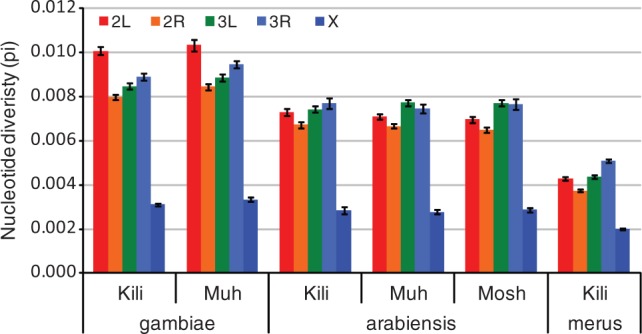
Nucleotide diversity (*π*) for each population and chromosome arm. From single species data sets 3, 4, and 5. Kili = Kilifi, Muh = Muheza, Mosh = Moshi.

Nucleotide diversity on the X chromosome is low in all populations and species (35–45% that of the autosomes, significant by *t*-test of moving window of autosomal vs. X chromosome π, *P* < 0.0001 for each species and for each population; fig. 7). X chromosome diversity is expected to be lower than the autosomes due to the fact that it spends less time in males (where a higher amount of mutation occurs due to the mechanism of gamete formation) and because of its lower effective population size (three-fourth the *N*_e_ of the autosomes). Using an estimate of the ratio of male to female mutation rate from *Drosophila miranda* (Bachtrog 2008), we would expect our X chromosome diversity to be approximately 67% autosomal. The additional reduction in diversity could be due to a higher male mutation rate in *Anopheles* than *Drosophila* or selection. In all species, our RADseq reads mapped disproportionately to exons on the X chromosome, so this will also create an underestimate of its average diversity.

The pattern of nucleotide diversity can be used to infer aspects of the long-term demography of the populations, and in particular, deviations from the null model of neutral variation in an isolated population of constant size. To examine this question, we first calculated Tajima’s *D* statistic, the standardized difference between *π* and *θ*_W_ (Tajima 1989). Negative Tajima’s *D* (*π* < *θ*_W_; an excess of rare alleles) indicates population expansion, purifying selection, or selective sweeps, whereas positive Tajima's *D* (low levels of both low- and high-frequency polymorphisms) indicates a decrease in population size and/or balancing selection (a feature of the maintenance of segregating inversions [reviewed in Andolfatto et al. 2001]). Whole-genome Tajima’s *D* and per chromosome arm values for each population are shown in table 2 and supplementary table S3, Supplementary Material online. All *A. arabiensis* populations and *A. merus* had significantly negative Tajima’s *D* (*t*-test across chromosome arms, *P* < 0.05), whereas *A. gambiae* from Kilifi was significantly positive (*P* = 0.0079) and from Muheza was mostly negative but nonsignificant (*P* = 0.082). Pooled *A. arabiensis* (from all locations) had an overall Tajima’s *D* that was more negative than the any of the three individual sampling locations (table 2). This may seem surprising but is consistent with models of population growth in a structured, nonpanmictic population (Städler et al. 2009) and demonstrates that even when gene flow is high (as measured by *F*_ST_) demographic factors such as population expansion may be underestimated if all samples are drawn from a single deme. For the pooled *A. gambiae* data, Tajima’s *D* falls between the values for each sample site, both for the whole-genome data and consistently across chromosome arms (data not shown). The only significant Tajima’s *D* in the pooled data is a positive value for 2L (which is also positive for each sample site), most likely due to balancing selection acting on the segregating 2La inversion. The two sampling locations have significantly different Tajima’s *D* (*t*-test across chromosome arms, *P* = 0.0014). The positive Tajima’s *D* across the genome for the Kilifi samples reflects a deficit in low-frequency polymorphisms, and as this is a genome-wide effect it is more likely to be due to demography than selection, suggesting a recent decline in population size.

We also looked at the (folded) allelic frequency spectrum (AFS) to find departures from neutrality using a diffusion approximation method in *dadi* (Gutenkunst et al. 2009). For each population, a model incorporating growth gave a significantly better fit than the standard neutral model. For *A. arabiensis* and *A. merus* populations, the best models included one growth step (either instantaneous or exponential, supplementary table S4, Supplementary Material online). The optimized growth and time parameters for the best-fitting models are shown in table 3. In all cases, the amount of growth is small (between 1.76 and 4.77 times the ancestral population size). For both *A. gambiae* populations, the best *dadi* demographic model was bottle_growth (instantaneous size change with exponential recovery). In both cases, the instantaneous size change was a large increase in size (rather than a decrease as is usually associated with a bottleneck), and recovery consisted of a subsequent decrease in population size (see supplementary fig. S14, Supplementary Material online, for illustration of models). Due to small sample sizes, confidence intervals on the actual parameter values are large in most cases, but we can infer that 1) both *A. gambiae* populations fit a model where there has been a substantial increase of the population in the past and more recent population shrinkage; and 2) this model is different from the best model for *A. arabiensis* and *A. merus* populations which instead show a small level of growth. A previous study of *A. gambiae* and *A. arabiensis* in East Africa using mtDNA and microsatellites observed that *A. gambiae* showed higher departures from mutation–drift equilibrium than *A. arabiensis* (Donnelly et al. 2001). Our results show that this is also true of genome-wide data and is consistent across different sample sites.

**Table 3.** Demographic Models with Highest Log Likelihood and Optimized Parameters.

Species	Pop	Best Model^a^	Log Likelihood	Final Pop Size^b^ (95% CI)	Bottleneck Size^b^ (95% CI)	Time^c^ (95% CI)
*A. gambiae*	Kilifi	Bottle_growth	−41.57	0.70 (0.34–1.79)	129.75 (20.91–338.60)	1.21 (0.20–3.39)
	Muheza	Bottle_growth	−36.91	2.78 (0.94–5.17)	118.46 (8.89–499.87)	3.63 (0.83–6.25)
*A. arabiensis*	Kilifi	Two_epoch	−40.51	4.77 (2.01–6.08)		5.24 (1.18–7.45)
	Muheza	Exp_growth	−39.53	1.79 (1.59–99.98)		0.56 (0.02–7.64)
	Moshi	Two_epoch	−37.33	1.95 (1.58–3.72)		0.421 (0.11–2.95)
*A. merus*	Kilifi	Two_epoch	−38.73	1.76 (1.39–54.98)		0.18 (0.03–1.70)

^a^See supplementary fig. S13, Supplementary Material online.

^b^Relative to ancestral population size.

^c^From start of growth to present, units 2*N*_e_ generations. SNP sets 3, 4, and 5, one random SNP per tag location, autosomes only, segregating inversions removed.

Although they have the same best optimal model, there is a substantial difference in parameter values between the *A. gambiae* samples from Kilifi and Muheza. The final (present day) Kilifi population size is lower than the ancestral size, whereas Muheza shows a slight increase (this was also consistent in the second best model of exponential growth). In addition, the Kilifi expansion is much more recent than that of Muheza. As we have already stated, the actual parameter values should be treated with caution, but taken together with the genome-wide positive Tajima’s *D* and significantly lower diversity in the Kilifi sample, the two populations appear to have different demographies.

### Assessing Possible Biases Introduced by RADseq

RADseq involves sequencing the DNA immediately on both sides of a restriction cut site, therefore heterozygous polymorphisms at cut sites, and two cut sites close together, will result in a systematic underestimate of diversity (Luca et al. 2011). Simulations have shown that using only sites that are present in every sample gives close to the “true” diversity parameters at those loci (Arnold et al. 2013). However, this means that loci with particularly high diversity will not be included in the analysis, so creating a slight downward bias in the overall diversity. Therefore, we expect all our diversity measures to be underestimates of the true values. In addition, we have observed in our data that the number of informative sites decreases considerably as more species are added to the alignment (with RAD tags included only if they are present in every sample), and that there is an accompanying small decrease in π across all species, even in *A. gambiae* (supplementary fig. S13, Supplementary Material online). This is because adding more samples and species to a data set means that cut sites that are common to all samples are skewed toward exons (constrained by purifying selection), indicated by an increase in the proportion of exonic sites (supplementary fig. S13, Supplementary Material online). In addition, the *A. merus* only data set (data set 5) shows a similar increase in exonic sites due to the divergence of *A. merus* from the PEST reference. If we remove the exonic sites from the data sets (leaving only sites in putatively neutral, unconstrained regions of the genome), then diversity slightly increases, but neither relative diversity of *A. gambiae*, *A. Arabiensis*, and *A. merus* nor the ranking of populations within species changes (supplementary fig. S13, Supplementary Material online).

When measuring divergence between species or populations using any sequence data (not just RADseq), we can only use sequences that are orthologous between samples and therefore cannot use the most divergent regions of the genome. In RADseq, this effect is exacerbated by the fact that some cut site polymorphisms will be fixed between species or populations. However, simulations have showed that restricting analysis to loci where there is no missing data results in an *F*_ST_ distribution very similar to the true distribution (Arnold et al. 2013), so in each of our data sets we have only included loci that are present in every sample.

### Conclusions and Implications for Vector Control

The *A. gambiae* species complex is genetically heterogeneous, with multiple sibling species and molecular forms, some only recently discovered, and it is by no means certain that the current list of taxa is complete (e.g., Riehle et al. 2011). In this article, we have used the method of RADseq to sequence up to 253,000 bp distributed across the mosquito genome. Such data should be enough to detect cryptic species, but none were found in our sample. It is important for planning and monitoring of vector control to be aware of the species that are present. RADseq will be a useful method for detecting and delineating otherwise cryptic taxa.

Although global analyses of the multilocus RADseq data clearly distinguished the three species included in our study, we also found abundant shared polymorphism among the three species, particularly between *A. gambiae* and *A. arabiensis*, where they substantially outnumber fixed differences. This finding reinforces the idea that species in the complex are closely related to each other. One implication of this is that the species may share standing variation at loci that potentially confer important phenotypes such as insecticide resistance, meaning that they could respond in similar ways to selective pressures. Our finding that *A. merus* is the most diverged of the three species contradicts the traditional phylogeny based on chromosomal inversions but is consistent with a recently revised chromosomal phylogeny (Kamali et al. 2012).

Absolute levels of divergence between *A. gambiae* and *A. arabiensis* are more or less consistent across the genome, with the exception of the region covered by the Xag inversion, where divergence is substantially higher. The simplest explanation for the higher divergence in Xag is that gene flow between the species stopped first in this region, due to reproductive incompatibilities caused by one or more genes within the inversion, and continued for longer in other genomic regions. We did not find evidence that the 2La inversion has introgressed between the species more often or more recently than other autosomal regions. Whether gene flow is still going on between *A. gambiae* and *A. arabiensis* in East Africa is unclear. Further genomic analysis of a more geographically extensive sample including *A. coluzzii* may help to clarify this issue, which is of obvious importance for the spread of insecticide resistance and transgenes between species.

Our RADseq analysis was also able to identify chromosomal inversions segregating in the different species as islands of increased LD. The *A. gambiae* species complex has been subject to extensive cytological surveys, and so, not surprisingly, we did not detect any new inversions. In species that have not received such extensive cytological surveys, RADseq and LD analyses will allow inversions to be documented for the first time. The balancing selection that maintains polymorphic inversions within populations and species has a dramatic effect on the genealogy of loci within the inversions, and this must be taken into account (or such loci excluded from) population genomic analyses of demographic history.

Population differentiation over the scale of the study (∼190–270 km) was weak in both *A. gambiae* and *A. arabiensis*, consistent with recent movement among populations. The high number of genome-wide markers provided by RADseq gives more confidence in measurements of population differentiation than could be provided from previous more limited studies. RADseq can also provide baseline data on genomic diversity, which may be important when monitoring the impact of vector control efforts. Baseline data can also be used for parameterizing models, which aid in the design and implementation of vector control strategies. Analyses of Tajima's *D* and site frequency spectrum suggest that *A. gambiae* has a more complex demographic history than *A. arabiensis* or *A. merus*, which will need to be taken into account in future genomic analyses, such as analysis of selection acting upon specific loci and demographic effects of vector control efforts.

## Methods and Materials

### Sample Collection and Preservation

Mosquitoes were collected at three locations in East Africa: Muheza and Moshi in Tanzania and Kilifi District in Kenya (fig. 6). Most mosquitoes were collected by indoor light trap, with the exception of three *A. arabiensis* in the final sample set which were collected by aspiration. *Anopheles gambiae* and *A. arabiensis* were collected in May, June, and July 2010; *A.* merus were collected in October 2009 and May 2010. All mosquitoes were preserved in 80% ethanol. Mosquitoes collected by aspiration were kept alive in the laboratory for 2 days to allow blood meals to digest before preservation.

### DNA Extraction, Species Identification, and Quality Control

DNA was extracted from individual *A. gambiae s.l.* mosquitoes using the DNeasy Blood and Tissue kit (Qiagen), by the standard protocol including an RNase digest and two 200 µl elutions. Each DNA sample was concentrated by ethanol precipitation. Each sample was identified to species level using an allele-specific PCR based on rDNA (Fanello et al. 2002). DNA quality and quantity were assayed by picogreen assay (Quant-It) and agarose gel electrophoresis.

### RAD Library Construction and Sequencing

DNA from individual mosquitoes was sent to Floragenex (Oregon) for RAD library construction and sequencing. For details of RAD sequencing, see Baird et al. (2008). Briefly, RAD sequencing employs Illumina short-read sequencing on a small but reproducible portion of the genome, by first digesting genomic DNA with a restriction enzyme and then using special adapters so that only DNA immediately on each side of the cut site is sequenced. The number of cut sites and therefore RAD tag sequences can be predicted from the reference genome (if one is available) or mathematically based on the genome size. We used Sbf1 restriction enzyme, which is a six base symmetrical cutter and which would give a predicted 3,800 cut sites in the *A. gambiae* genome. The single-read RADseq libraries were sequenced on an Illumina GAIIx.

### SNP Discovery and Genotyping

#### Preprocessing of Raw Reads

We preprocessed the raw Illumina reads into pools for each mosquito sample, using RADtools v1.1.1 (Baxter et al. 2011). The fuzzy MIDs option was used which accepts reads with an error in the MID (Molecular Identifier or barcode) and assigns them to the nearest pool; if the MID can be assigned to more than one pool, a new pool will be created and named after all the possible pools for the ambiguous MID. The original Illumina data consisted of 45,711,425 reads. 41,224,668 reads were pooled into 72 sample files after 4,057 (<0.01%) reads were discarded as they did not contain a recognizable MID, and 4,482,700 (9.8%) reads were discarded due to lack of the restriction site overhang. No ambiguous pools were created.

#### Alignment to Reference

We aligned the preprocessed reads from each sample to the *A. gambiae* PEST reference genome in BWA v0.5.9 (Li and Durbin 2009). No seed was used, and maximum edit distance was set to 0.01 (equating to 5/64 mismatch bases) for *A. gambiae* and 0.001 (equating to 7/77 mismatched bases) for all other species to allow for some sequence divergence from the PEST reference. The Burrows–Wheeler alignment algorithm chooses the alignment with the fewest mismatches; if two alignments are equally good, one is chosen randomly and the alignment is flagged as a “repeat.” A mapping quality score is assigned to each alignment that is scaled according to the phred score of the mismatched bases, the difference between the best hit and second best hit, and the number of equally good second best hits. We removed all repeat alignments from the final alignment files.

#### Variant Genotyping

We combined the alignments from each sample into a single file using mpileup in SAMtools v0.1.18 (Li et al. 2009). Bases with a phred quality score of less than 25 were excluded, and probabilistic BAQ realignment was switched off. This option can result in false-positive SNPs around indels, so in a subsequent filtering step we removed SNPs 6 bp or less around an indel. Variant sites were called using bcftools. Variants sites were filtered to remove all sites where any samples had less than 15× coverage. All indels and multiallelic sites were removed from the data set. A random set of the final SNPs were checked against the original alignment files using IGV to make sure that they were being called correctly. We also used bcftools to output genotypes at all sites, which were filtered as above to give data sets containing both variant and invariant sites. We performed variant and genotype calling for 1) all samples; 2) *A. gambiae s.s* and *A. arabiensis* samples; 3) *A. gambiae* samples; 4) *A. arabiensis* samples, and 5). *A. merus* samples. VCF files for invariant and variant sites for each species have been deposited in Dryad Digital Repository: doi:10.5061/dryad.hm6tt.

Most RADseq tags aligned in pairs on either side of a restriction site giving an alignment of ∼144 bp. These pairs of RADseq tags are closely linked and so are considered in all analyses as one chromosomal location referred to below as the “tag location.” To avoid bias caused by close linkage, all analyses except those including invariant sites (i.e., *D*_XY_ and diversity measures) and LD (i.e., *r*^2^) were carried out on SNP data with one SNP randomly selected per tag location and were repeated using at least three such random SNP sets.

### Analysis of SNPs Obtained from RAD Tag Sequences

#### Identification of Breeding Units and Relationships between the Three *A. gambiae s.l.* Species

We used three approaches to look for cryptic reproductive units that would not be identified by the rDNA identification method. 1) We created an unrooted neighbor-joining phylogeny using average pairwise distances (*D*_XY_ from ARLEQUIN v3.5.1.2 [Excoffier and Lischer 2010]) as the distance matrix, for both autosomal and X chromosome SNPs (PHYLIP v3.69). 2) We used the clustering software STRUCTURE v2.3.2 (Pritchard et al. 2000), the “admixture” model with correlated allele frequencies, and 10^6^ burn-in steps and 10^6^ simulations. We simulated 1–5 populations (*k* = 1 to *k* = 5). 3) We used plots of the first and second principal components to visualize variation in the SNP data, for autosomal and X chromosome SNPs separately (principal components based on correlations carried out in JMP v10.0.0). For all subsequent analysis, we used the species defined by the analyses above. Fixed nucleotide differences and shared and private polymorphisms were counted between species, overall and in a sliding window across the genome. We measured differentiation between each species both by pairwise *F*_ST_ (Weir and Cockerham 1984) in ARLEQUIN, tested for significance by 8,000 permutations, and by average and net pairwise differences (*D*_XY_ and *D*_A_; DNAsp v5 [Librado and Rozas 2009]). To avoid bias from closely linked SNPs, we measured *F*_ST_ using one randomly selected SNP per RAD tag location and repeated this five times with different random SNPs. We report the median of the five *F*_ST_ values. Whole-genome data was used unless otherwise stated (i.e., autosomal or X chromosome).

#### Population Structure

To test for structure within and between populations, we again used STRUCTURE v2.3.2 as above. We also used the randomized SNP data sets for principal components analysis on the whole genome and for SNPs within inversions (JMP v10.0.0, SAS Institute Inc., Cary, NC). A measure of migration (*N*_e_*m*) based on *F*_ST_ (from *N*_e_*m* = (1 − *F*_ST_)/4*F*_ST_) was calculated in ARLEQUIN. As above, we calculated pairwise *F*_ST_ between each population one random SNP per tag data sets and also plotted locus-by-locus *F*_ST_ across the genome for the two *A. gambiae* populations and the three *A. arabiensis* populations.

#### Inversions

We calculated the pairwise LD (*r*^2^) between all loci using Haploview v4.2. (Barrett et al. 2005). Triangle plots of LD across each chromosome for each population were made in Haploview. Where segregating chromosomal inversions were identified, we performed PCA of the SNPs inside the inversions and plotted the first and second principal components with each individual sized in proportion to its heterozygosity. We plotted *F*_ST_ in a sliding average across 2L for the *A. gambiae* 2La homokaryotypes and across 2R for the *A. arabiensis* 2Rb homokaryotypes. We also plotted *D*_XY_ across 2L between *A. arabiensis* and the two *A. gambiae* 2La homokaryotypes.

#### Nucleotide Diversity, LD, and Population History

We calculated standard diversity indices for each species overall and for each population for the SNP sets and for the data sets consisting of variant and invariant sites. We calculated the inbreeding coefficient *F*_IS_ for each population and locus-by-locus observed and expected heterozygosity from the SNP data sets. To look for variations in diversity across the genome, we calculated *π* in a sliding window of 1,000 bp in 10-bp steps on the variant and invariant data set, for each species and for the two *A. gambiae* populations separately. ARLEQUIN v3.5.1.2 was used for all analyses of the SNP data sets, and DNAsp v5 was used for all analyses of the variant and invariant data sets.

We calculated effective population size from *θ* = 4*N_θ_μ*, where *μ* is the mutation rate. No estimate of nuclear mutation rate is available for *Anopheles*, so we used the mutation rate 1.1 × 10^−^^8^ per year estimated from divergence of *Drosophila* lineages (Tamura et al. 2004) and assumed 10 generations per year.

We modeled different demographic scenarios using a diffusion-based approach in *dadi* (Gutenkunst et al. 2009), which calculates the likelihood of different demographic models given an observed allele frequency spectrum (AFS). Each species from each location was modeled separately; one SNP per tag location was used, and SNPs on the X chromosome and within polymorphic inversions were excluded. This resulted in data sets with the following number of segregating SNPs per population: 877 *A. gambiae* Kilifi, 1,016 *A. gambiae* Muheza, 769 *A. arabiensis* Kilifi, 814 *A. arabiensis* Muheza, 815 *A. arabiensis* Moshi, and 1,002 *A. merus.* An outgroup was not available for these species, so a folded spectrum was used, which ignores ancestral state information. For each folded AFS, we modeled increasingly complex single population demographic scenarios, which included instantaneous size change and exponential growth (or shrinkage). Because the distance between SNPs was large, we considered the SNPs as independent data and compared models by likelihood ratio test. Confidence intervals for the optimum parameters of the best models were calculated by nonparametric bootstrapping.

## Supplementary Material

Supplementary results, figures S1–S14, and tables S1–S4 are available at *Molecular Biology and Evolution* online (http://www.mbe.oxfordjournals.org/).

Supplementary Data
