# Supplementary material for: Genomic Analyses of Three Malaria Vectors Reveals Extensive Shared Polymorphism but Contrasting Population Histories
Source: Mol Biol Evol. 2014 Jan 9;31(4):889–902. doi: 10.1093/molbev/msu040 (PMC3969563; doi:10.1093/molbev/msu040)
Supplement: Supplementary Data [file supp_31_4_889__index.html]

Genomic Analyses of Three Malaria Vectors Reveals Extensive Shared Polymorphism but Contrasting Population Histories. — Genomic Analyses of Three Malaria Vectors Reveals Extensive Shared Polymorphism but Contrasting Population Histories — Genomic Analyses of Three Malaria Vectors Reveals Extensive Shared Polymorphism but Contrasting Population Histories — Supplementary Data 

# Genomic Analyses of Three Malaria Vectors Reveals Extensive Shared Polymorphism but Contrasting Population Histories

## Supplementary Data

files

**Files in this Data Supplement:**

- Supplementary Data - pdf file
- Supplementary Data - pdf file
- Supplementary Data - pdf file
