# Supplementary material for: Genomic Analyses of Three Malaria Vectors Reveals Extensive Shared Polymorphism but Contrasting Population Histories
Source: Mol Biol Evol. 2014 Jan 9;31(4):889–902. doi: 10.1093/molbev/msu040 (PMC3969563; doi:10.1093/molbev/msu040)
Supplement: Supplementary Data [file supp_msu040_SupplementaryFigures1to9ForMBEresubmission.pdf]

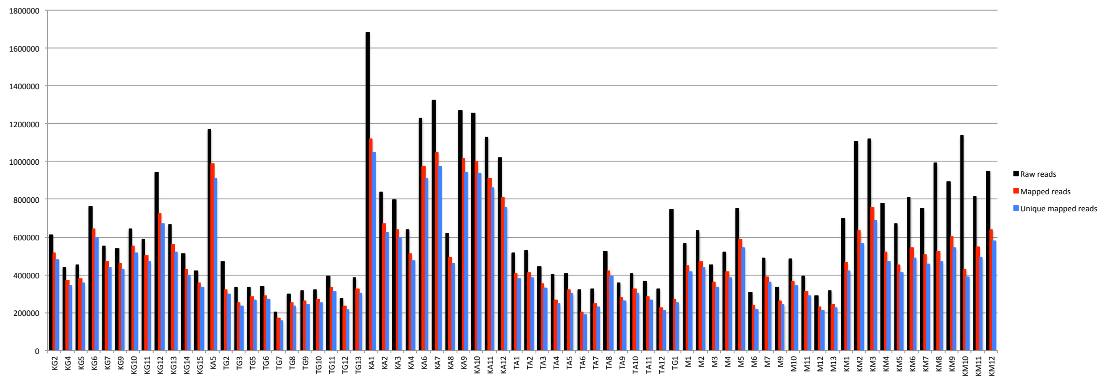

Supplementary Figure S1. Number of RADseq reads per sample. Black=raw reads; red=reads mapped to PEST; blue=reads uniquely mapped to PEST.

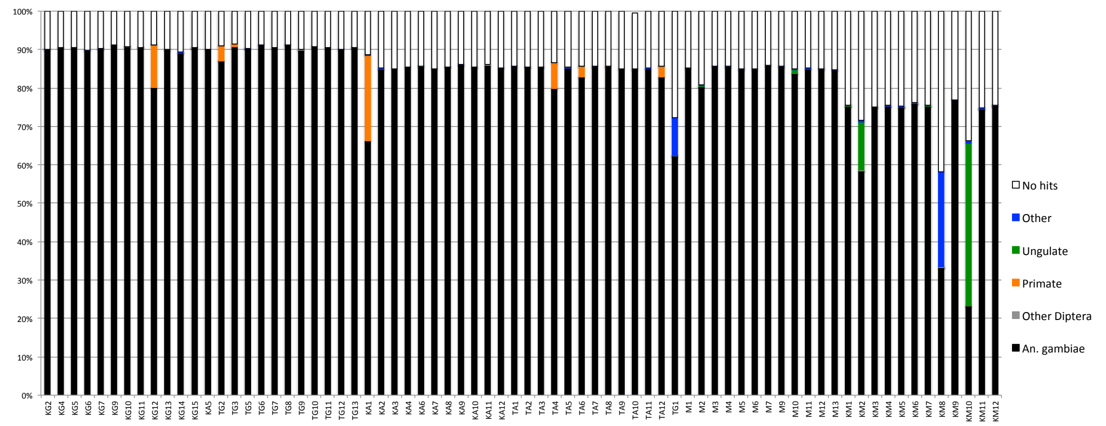

Supplementary Figure S2. BLAST results for raw RADseq reads, using the top BLAST hit for each read.

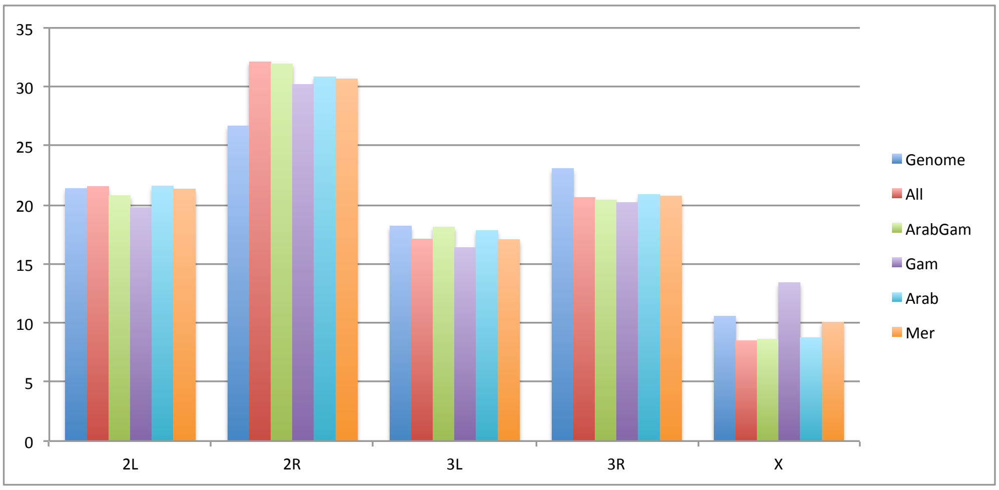

Supplementary Figure S3. Chromosomal location of RADseq reads. Y-axis=percent of total. The Genome bar refers to the percentage of the genome of each chromosome in the PEST reference.

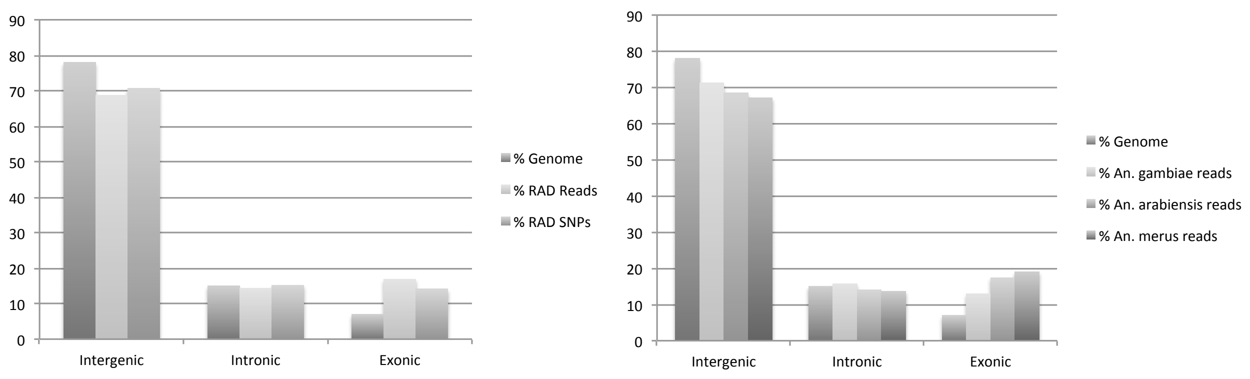

Supplementary Figure S4. Location in genome of RADseq reads and SNPs. % Genome refers to percentage of the genome that is intergenic, intronic and exonic in PEST.

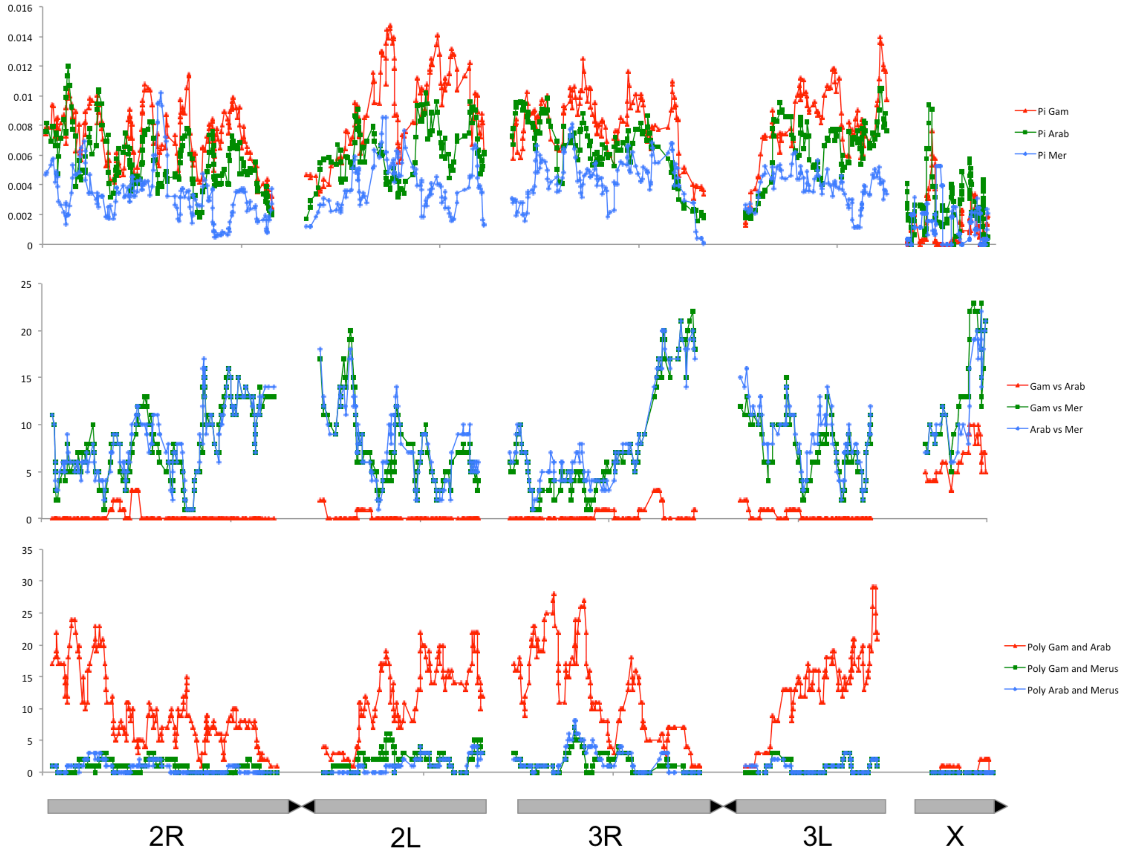

Supplementary Figure S5. Nucleotide diversity (top), fixed differences (middle) and shared polymorphisms (bottom) across the genome.

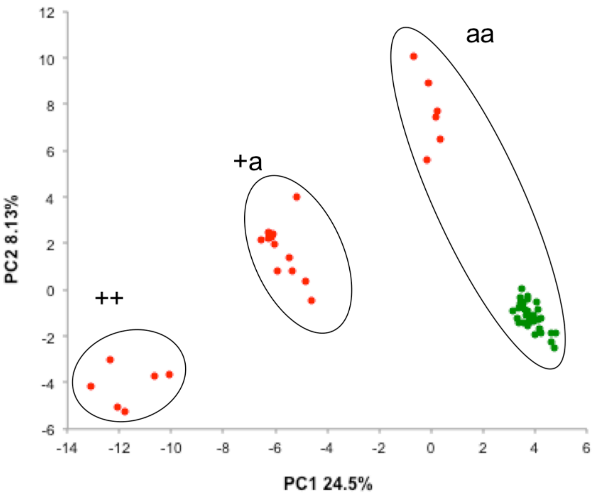

Supplementary Figure S6. Principal components analysis of 126 SNPs within 2La. Red= *An. gambiae*, Green= *An. arabiensis.* SNP set 2.

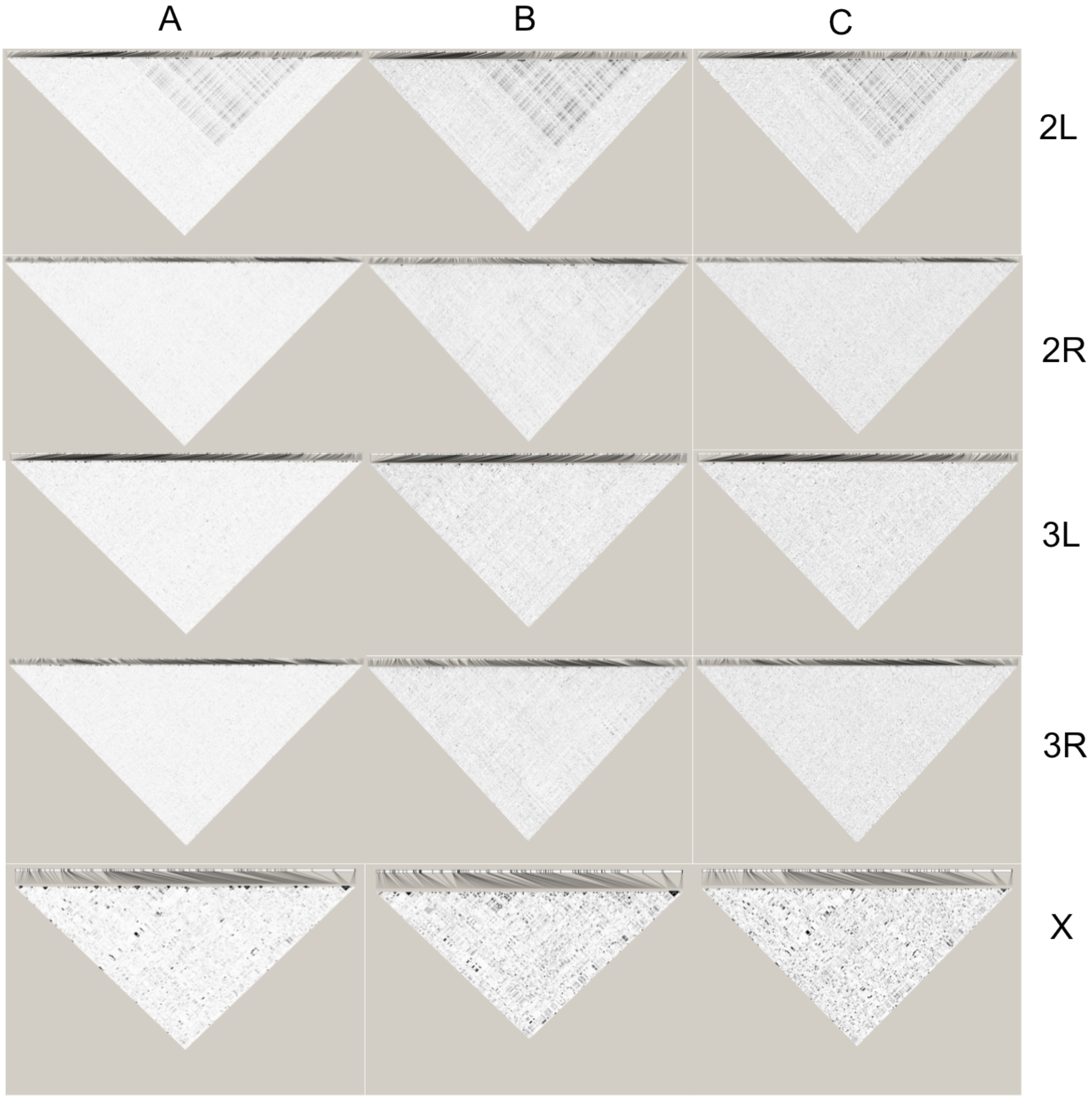

Supplementary Figure S7. LD plots for each *An. gambiae* chromosome arm. A: all *An. gambiae* samples; B: *An. gambiae* from Kilifi; C: *An. gambiae* from Muheza.

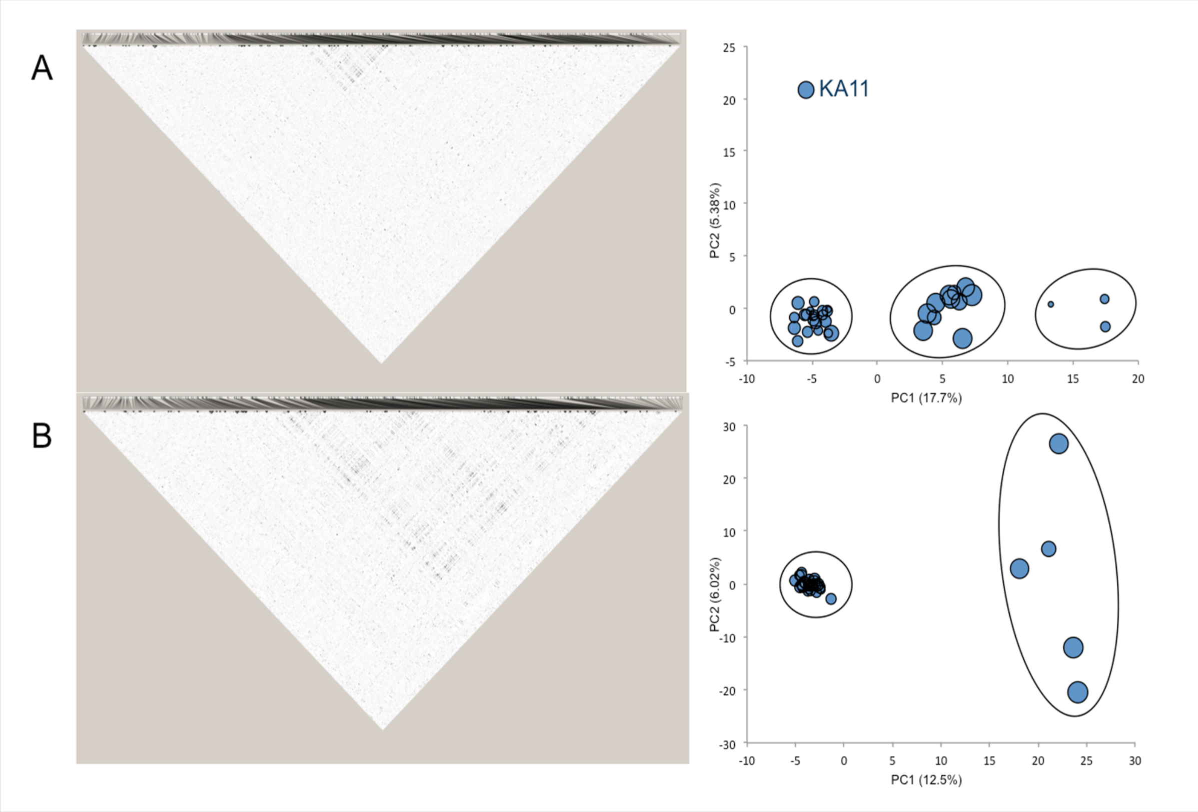

Supplementary Figure S8. LD grid plots and principal components analysis of *An. arabiensis*. A: LD grid plot of chromosome arm 2R and PCA plot from 306 SNPs within the 2Rb inversion; B: LD grid plot of chromosome arm 3R and PCA plot from 704 SNPs within the 3Ra inversion. LD grid plots show pairwise linkage disequilibrium between each pair of markers across the chromosome, and are shaded by r-squared values; light for low r-squared (no linkage) to black for total linkage. The circles in the PCA plots are scaled to the level of expected heterozygosity for each sample. SNP set 4.

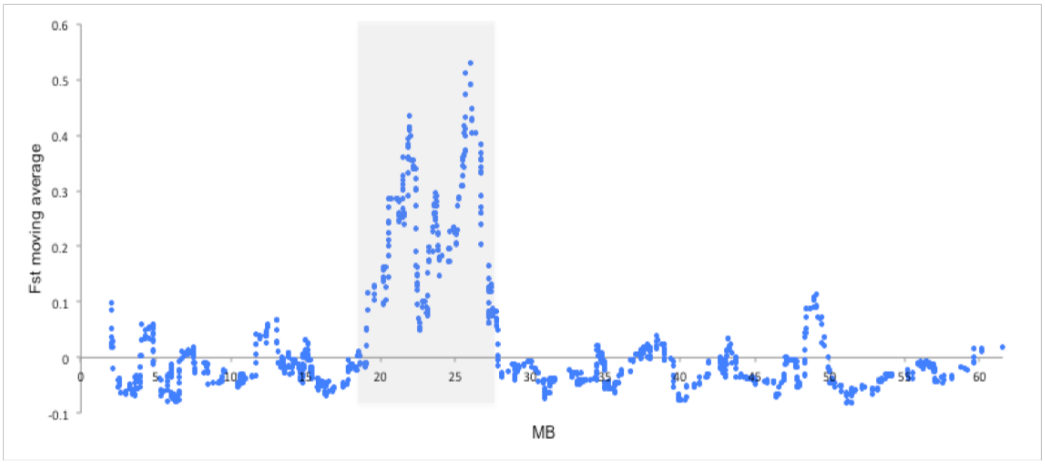

Supplementary Figure S9. F_{ST} between *An. arabiensis* 2Rb homokarytype samples, across chromosome 2R. Shaded area denotes location of 2Rb inversion. F_{ST} measured locus-by-locus and averaged in a 25 SNP window moving in 1 SNP steps. Average F_{ST} within 2Rb =0.452. SNP set 4.
