# Supplementary material for: Genomic Analyses of Three Malaria Vectors Reveals Extensive Shared Polymorphism but Contrasting Population Histories
Source: Mol Biol Evol. 2014 Jan 9;31(4):889–902. doi: 10.1093/molbev/msu040 (PMC3969563; doi:10.1093/molbev/msu040)
Supplement: Supplementary Data [file supp_msu040_SupplementaryFigures9to14ForMBEresubmission.pdf]

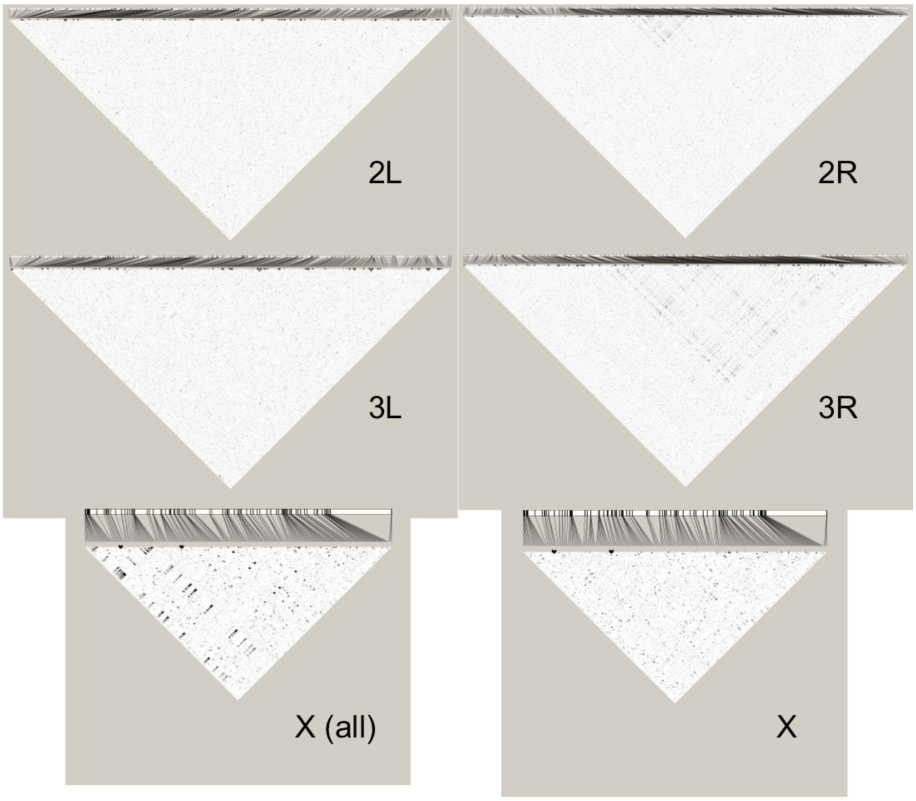

Supplementary Figure S10. LD plots for each *An. arabiensis* chromosome arm. X (all) includes sample KA11.

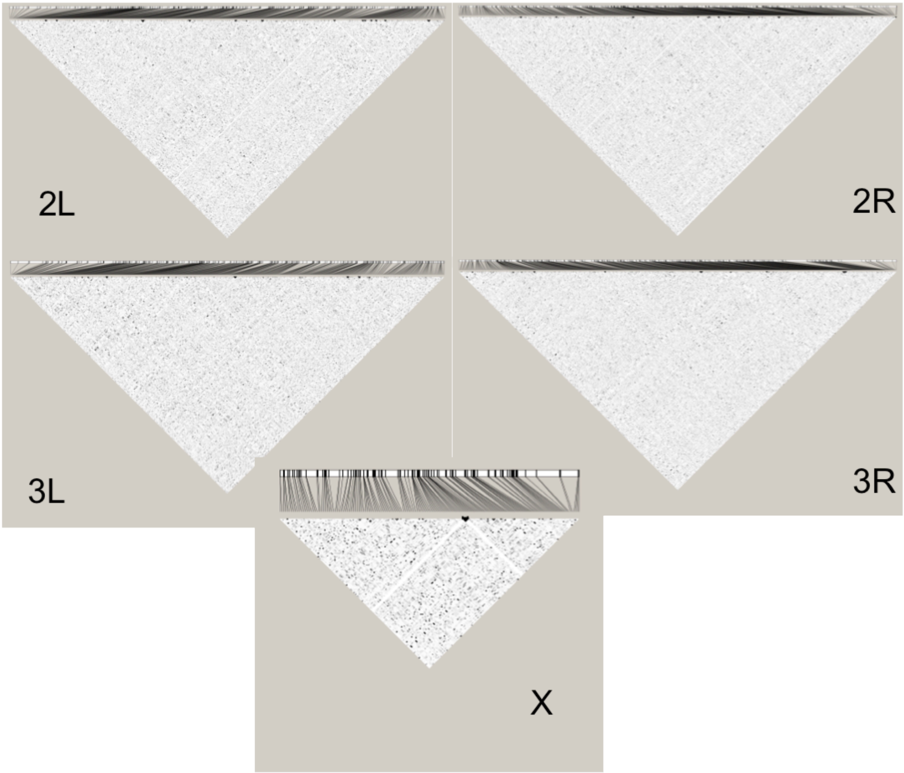

Supplementary Figure S11. LD plots for each *An. merus* chromosome arm.

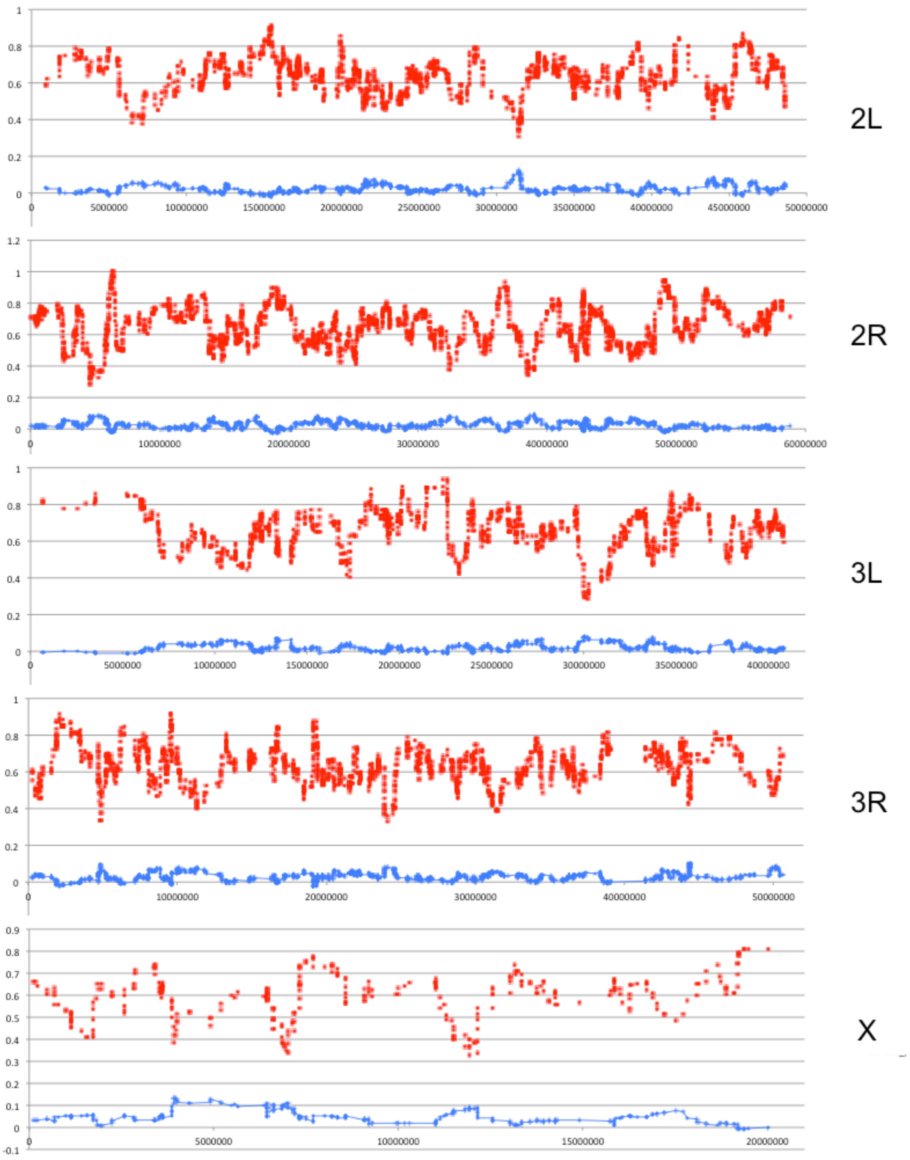

Supplementary Figure S12. Fst (blue line) and p-value (red line) between *An. gambiae* populations. Sliding window of 25 SNPs in 1 SNP steps. P-value from 8000 permutations.

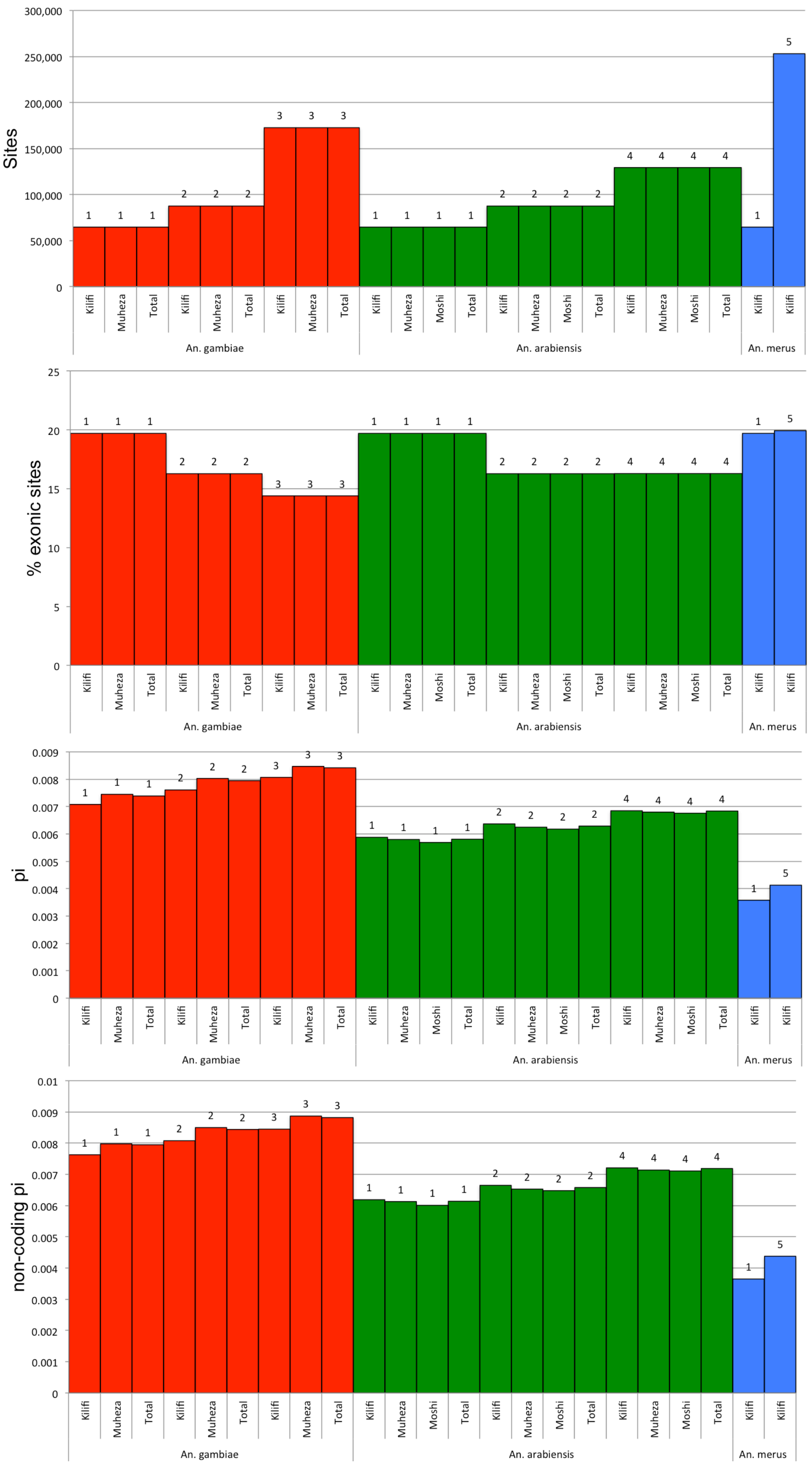

Supplementary Figure S13. Number of sites, percent exonic sites, nucleotide diversity (pi), and non-coding pi for each species, for each data set described in Table 1. Each column is labeled with the number of the corresponding data set.

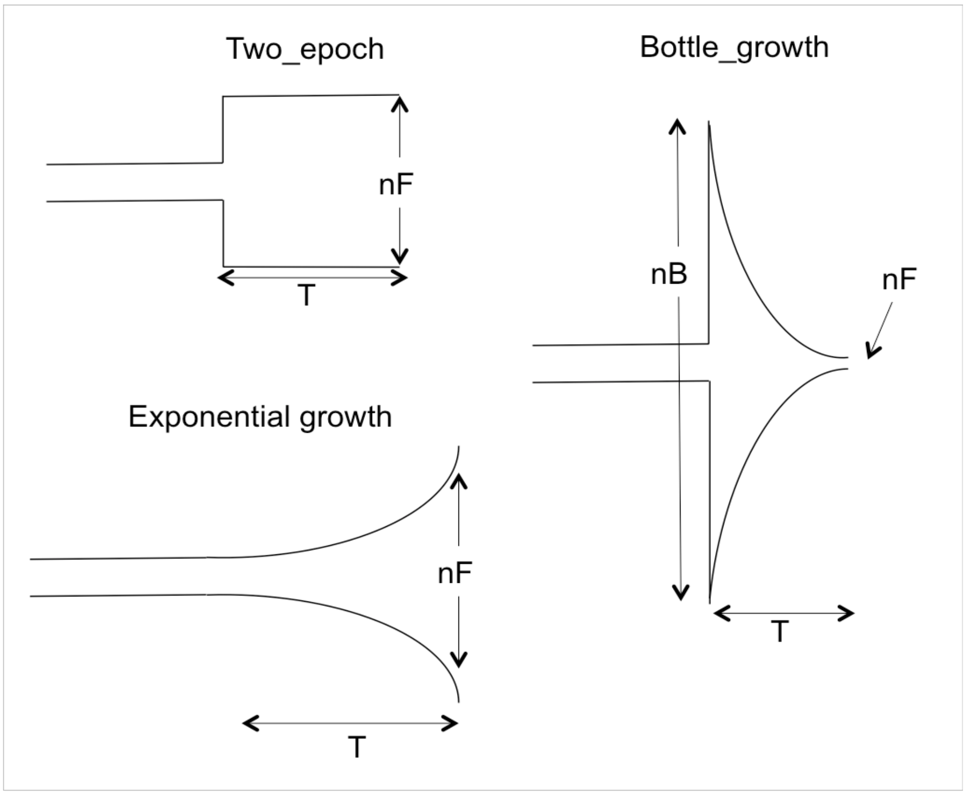

Supplementary Figure S14. The three best *dadi* demographic models. Two_epoch: one instantaneous size change time T ago to nF (final population size). Exp_growth: Exponential growth (or shrinkage) starting time T ago, resulting in final populations size nF. Bottle_growth: Instantaneous size change time T ago, followed by exponential growth (or shrinkage) to final size nF.
