# Supplementary material for: Genomic Analyses of Three Malaria Vectors Reveals Extensive Shared Polymorphism but Contrasting Population Histories
Source: Mol Biol Evol. 2014 Jan 9;31(4):889–902. doi: 10.1093/molbev/msu040 (PMC3969563; doi:10.1093/molbev/msu040)
Supplement: Supplementary Data [file supp_msu040_SupplementaryResults_and_TablesForMBEresubmission.pdf]

Supplementary Results.

Collection summary

Mosquitoes from the three collection locations were identified based on rDNA (Fanello et al. 2002). *An. gambiae* was found only at Kilifi and Muheza; *An. arabiensis* was found at all three sites; *An. merus* was found only at Kilifi. No other members of the *An. gambiae* complex were found. The proportions of *Anopheles* species found at each site are shown in Table S1.

RAD-sequencing

Sbf1 RAD sequencing libraries were successfully created for 72 samples and sequenced on the Ilumina GAIIx. The number of reads per sample averaged 634,880±316,963 (sd; Figure S1). Blast searches against all available reference genomes revealed that a high percentage of reads were from mosquitoes rather than microbial or blood meal contaminants, with the exception of two *An. merus* samples (Figure S2). In the BWA alignments against the *An. gambiae* reference PEST genome, 85% of *An. gambiae* reads aligned (79% uniquely). This figure dropped to 80% for *An. arabiensis* reads (75% uniquely) and 68% for *An. merus* reads (61% uniquely). The decrease presumably reflects divergence from the reference genome, rather than being contamination from non-*Anopheles* DNA. Mean depth of coverage in the BWA alignments was 34±49(sd), 40±65, and 95±83 for *An. gambiae, An. arabiensis,* and *An. merus,* respectively.

In silico RAD digestion of *Drosophila* showed that enzyme choice can have a significant impact on the location of RAD cut sites due to GC content (Arnold et al. 2013). In our data RAD-tags were spread across chromosome arms in proportion to their size (Figure S3), and were also from intergenic, intronic, and coding regions proportional to their size (Figure S4).

An. arabiensis sample KA11

An. arabiensis showed LD at several dispersed locations on the X chromosome (Figure S7), which do not correspond to any known polymorphic inversions. Further investigation revealed that one individual from Kilifi (designated KA11) was responsible for the LD, which disappeared when that sample was removed

from the analysis (Figure S7). PCA revealed that 25 SNPs on the X chromosome were heterozygous in KA11 and fixed in all other *An. arabiensis*. This individual was also an outlier in PCA of 2Rb SNPs (see Figure 7), where 28 SNPs were heterozygous in KA11 and fixed in all other *An. arabiensis*, and these also show up as a very small amount of the '*gambiae*' cluster in STRUCTURE analysis (Figure 1). In other analyses (PCA of other chromosome arms) KA11 was not differentiated from other *An. arabiensis* samples, and in 3Ra inversion analysis this sample fell in the main homokaryotype cluster. Where data was available, these sites were polymorphic in *An. gambiae*, segregating for the same two nucleotides as KA11. Discounting experimental error, it is possible that this *An. arabiensis* individual carried some *An. gambiae* alleles from a past hybridisation.

Micro-geographic population structure

Within each of the sites, *An. gambiae* and *An. arabiensis* were collected from a number of houses with maximum distances of 2-7km apart; the exception was *An. arabiensis* in Kilifi, which was collected from houses up to 32km apart. At this scale there was no significant correlation between the genetic distance between individuals (as measured by F_{ST} or D_{XY}) and geographical distance (Mantel test, $p>0.05$). Similarly, for *An. merus*, which was only collected in Kilifi, the 12 individuals were from two clusters 52km apart (n=6 in each), and F_{ST} was non-significant ($F_{ST}=0.005$, $p=0.22$). No clustering was found within *An. merus* (PCA and STRUCTURE, results not shown).

Fanello C, Santolamazza F, Della Torre A. 2002. Simultaneous identification of species and molecular forms of the *Anopheles gambiae* complex by PCR-RFLP. Med. Vet. Entomol. 16: 461-464.

Table S1. Composition of collections at each sampling location.

	Percentage composition of *Anopheles* species caught (N)[1]			Percentage of each *An. gambiae s.l.* species (N)[2]		
	An. gambiae s.l.	*An. funestus*	Other *Anopheles*	*An. gambiae*	*An. arabiensis*	*An. merus*
Kilifi, Kenya	69 (535)	28 (214)	3 (21)*	11 (20)	22 (38)	67 (118)
Muheza, Tanzania	100 (769)	0 (0)	0 (0)	80 (67)	20 (17)	0 (0)
Moshi, Tanzania	99 (534)	0 (0)	1 (4)**	0 (0)	100 (56)	0 (0)

[1]Percentage of total catch over collection period, morphologically identified; [2]Percentage of randomly selected sub-sample of *An. gambiae s.l.* identified by PCR; **An. pretoriensis*; ***An. pharoensis*.

Table S2 Comparison of locus-by-locus FST values for autosomes and X chromosomes.

	Mean Fst +/- SE		p-value (from t-test)
	Autosomes	X chromosome	
An. gambiae	0.0246+/-0.00197	0.0477+/-0.00989	0.0237
An. arabiensis	0.0043+/-0.00108	0.0044+/-0.00386	0.9848

Table S3. Genetic diversity by chromosome arm for all species and populations.

		An. gambiae[1]		*An. arabiensis*[2]			*An. merus*[3]
		Kilifi	Muheza	Kilifi	Muheza	Moshi	Kilifi
π x 10^{-3}	2L	10.07	10.33	7.31	7.08	6.96	4.30
	2R	7.98	8.45	6.73	6.67	6.50	3.74
	3L	8.48	8.87	7.43	7.73	7.71	4.35
	3R	8.91	9.46	7.69	7.46	7.66	5.09
	X	3.11	3.33	2.86	2.78	2.87	2.01
S	2L	1,120	1,216	773	789	772	909
	2R	1,431	1,677	1,021	1,077	1,040	1,128
	3L	887	999	664	722	706	784
	3R	1,156	1,307	846	879	900	1,079
	X	192	237	159	146	152	179
θ_W x 10^3	2L	9.01	10.24	7.85	7.65	7.65	4.43
	2R	8.96	369.79	7.21	7.27	7.17	3.96
	3L	9.24	328.70	8.07	8.38	8.38	4.85
	3R	9.89	263.86	8.57	8.51	8.90	5.39
	X	3.36	774.63	4.06	3.56	3.79	2.17
Tajima's D	2L	0.471	0.039	-0.279	-0.295	-0.365	-0.125
	2R	0.370	-0.235	-0.275	-0.325	-0.378	-0.224
	3L	0.329	-0.164	-0.325	-0.312	-0.320	-0.411
	3R	0.264	-0.180	-0.422	-0.492	-0.565	-0.222
	X	0.775	-0.041	-1.200	-0.862	-0.968	-0.288
Sites	2L	32,588	32,588	27,027	27,027	27,027	54,915
	2R	51,328	51,328	38,844	38,844	38,844	76,257
	3L	29,653	29,653	22,566	22,566	22,566	43,318
	3R	36,246	36,246	27,072	27,072	27,072	53,630
	X	19,348	19,348	10,735	10,735	10,735	22,113

[1]Data set 3; [2]Data set 4; [3]Data set 5.

Table S4. Log likelihoods for five demographic models.

Species	Pop	Standard neutral model	Two_Epoch	Exp_growth	Bottle_growth	Three_Epoch
		0 params	2 params	2 params	3 params	4 params
An. gambiae	Kilifi	-50.147	-44.894	-45.078	-41.565*	-41.379
	Muheza	-60.124	-42.577	-45.207	-36.908*	-36.887
An. arabiensis	Kilifi	-48.233	-40.512*	-41.049	-39.694	-39.585
	Muheza	-47.580	-39.716	-39.528*	-39.528	-38.810
	Moshi	-52.255	-37.325*	-37.329	-37.325	-37.325
An. merus	Kilifi	-48.424	-38.729*	-38.757	-38.682	-38.646

N. B. More complex models were tested but none resulted in improved log likelihood, so results are not shown. *Best fitting models for each population. SNP sets 3, 4 and 5, one random SNP per tag location, autosomes only, segregating inversions removed.
